# Systematic methodological flaws in DNA contamination assessment of mRNA vaccines: A critical analysis of Achs et al. (2025)

**DOI:** 10.18632/oncotarget.28913

**Published:** 2026-08-14

**Authors:** Kevin McKernan, David J. Speicher, Jessica Rose

**Affiliations:** ^1^Medicinal Genomics, Beverly, MA 01915, USA; ^2^Cyrus Scientific Inc, Hamilton, Ontario, Canada; ^3^Brownstone Institute, Austin, TX 78741, USA

**Keywords:** mRNA vaccines, DNA contamination, qPCR, plasmid DNA, RNA:DNA hybrids

## Abstract

Recent work by Achs et al. (2025) claimed to demonstrate no excessive residual DNA in COVID-19 mRNA vaccines using four orthogonal approaches. Since this work is being cited by regulatory authorities to claim DNA levels in mRNA vaccines are within limits, it is imperative that a robust public discussion occur regarding the methods strengths and weaknesses. We present a comprehensive critique identifying critical methodological flaws that systematically bias results toward underestimation of residual DNA contamination. These include: (1) inappropriate plasmid molecular weight calculations producing 1.5–10× underestimation (plasmid equivalents), (2) fragment size bias in primer design favoring detection of non-functional sequences over spike-encoding DNA, (3) thermal degradation of samples during library preparation, (4) analytical detection limits exceeding regulatory thresholds, (5) platform limitations excluding detection of kilobase-length DNA fragments, and (6) failure to account for RNA:DNA hybrid formation protecting spike DNA from degradation. Collectively, these flaws render the study's conclusions invalid for regulatory assessment of residual DNA contamination.

## INTRODUCTION

The presence of residual plasmid DNA in mRNA vaccines has emerged as a critical quality control and safety concern [1–3]. Regulatory guidelines establish limits of 10 ng DNA per dose [4], yet independent analyses have reported DNA levels exceeding these thresholds by orders of magnitude [5–12].

Beyond quantity, the quality and sequence composition of residual DNA raises specific oncogenic concerns. The production plasmids used in nucleoside modified mRNA vaccine manufacturing contain SV40 (Simian Virus 40) regulatory elements, including the SV40 promoter, two tandem 72 base pair enhancer sequences, a SV40 origin of replication and a ColE1/pMB1origin known to be a cryptic mammalian promoter. These elements pose multiple mechanistic risks:

- The SV40 enhancer contains a nuclear targeting sequence that facilitates DNA nuclear import [13].
- The SV40 promoter binds and sequesters p53, a critical tumor suppressor [14].
- The SV40 enhancer functions as a somatic hypermutation-targeting element, recruiting APOBEC3/AID cytidine deaminases that cause C-to-T point mutations and are a frequent source of lymphomagenesis [15].
- SV40-based plasmids demonstrate genomic integration capability [16].
- The residual DNA activates the cGAS-STING innate immune pathway [17], which has been mechanistically linked to myocarditis [18].
- The ColE1/pMB1 bacterial origin of replication is a known mammalian cryptic promoter and it resides adjacent to the Spike sequence [19].

These oncogenic risks were explicitly acknowledged by Moderna in their own patents. Moderna states that “residual DNA in drug products may induce activation of the innate response and has the potential to be oncogenic in patient populations” [20]. Moderna’s patents further note that “introduced DNA can integrate into host cell genomic DNA at some frequency, resulting in alterations and/or damage to the host cell genomic DNA” and that, “alternatively, the heterologous deoxyribonucleic acid (DNA) introduced into a cell can be inherited by daughter cells (whether or not the heterologous DNA has integrated into the chromosome) or by offspring” [21, 22].

Given these documented concerns, accurate quantification of residual DNA is essential for vaccine safety assessment. A recent publication by Achs et al. [23] claimed to refute reproduced independent findings of excessive residual DNA using “systematic analysis” with “four orthogonal approaches,” concluding no excessive DNA impurities exists in the Moderna and BioNTech COVID-19 mRNA vaccines.

However, detailed examination of their methodology reveals systematic biases that invariably underestimate the residual DNA detected in the vaccines tested. This critique documents specific technical flaws that invalidate their conclusions and their “safe” assessment regarding associated oncogenic risks.

## CRITICAL METHODOLOGICAL FLAWS

### Invalid plasmid molecular weight calculations

Achs et al. converted qPCR copy numbers to mass using the molecular weight of intact plasmids (4.92 × 10^6^ g/mol for the 7,569 bp Comirnaty plasmid), despite their own data showing median fragment sizes of 130–201 bp. While using the full plasmid molecular weight is theoretically acceptable, it requires applying fragmentation correction factors to account for the fact that random DNA breakage disrupts many qPCR amplicon targets. Achs et al. failed to apply any such corrections, resulting in an underestimation of residual DNA loads. It is also unclear from Achs et al., what is used in their equation to convert qPCR to nanograms. The equation speaks to “MW is the molecular weight of the fragment in grams per mole (based on its length in base pairs).”

Calculation of the error: There are three different numbers the reader could justify for fragment lengths:

- 63 bp KAN amplicon MW: 40,950 g/mol.
- 150 bp median fragment MW: 97,500 g/mol.
- 7,569 bp full length plasmid equivalents MW: 4.92 × 10^6^ g/mol.

Use of the wrong fragment length in their equation could lead to:

- Underestimation factor for 63 bp KAN amplicon: 4.92 × 10^6^/40,950 ≈ 120-fold.
- Underestimation factor for 150 bp median: 4.92 × 10^6^/97,500 ≈ 50-fold.

This error alone could account for their failure to detect contamination levels reported by other laboratories. The plasmid equivalents approach is fundamentally invalid when applied to fragmented DNA, as each detected fragment represents only a small portion of the original plasmid mass and the mathematical extrapolation from amplicons to full length plasmids rests on the assumed uninform digestion of the plasmid by DNase I.

### Plasmid size discrepancy

An additional inconsistency exists in the plasmid size used for calculations by Achs et al., who appear to have used 7,569 bp (MW = 4.92 × 10^6^ g/mol) based on reverse calculation from their reported 8 ng result for the KAN amplicon. The plasmid size used by Achs et al. is not explicitly documented in the study.

However:

- McKernan et al. measured 7,810 bp using Illumina and Oxford Nanopore sequencing [5].
- EMA regulatory documents disclosed by Pfizer specify 7,824 bp [24].
- Pfizer’s measurement likely represents the most accurate value due to their careful characterization of the poly-A tail length, which is difficult to resolve accurately on both Illumina and Oxford Nanopore platforms.

This 3.4% size discrepancy (7,569 vs. 7,824 bp) introduces an additional error in their mass calculations, compounding the fundamental molecular weight error described above.

### The missing fragmentation correction

Using full plasmid molecular weight is theoretically acceptable - but only if one applies proper fragmentation correction factors. When DNA is fragmented to median sizes of 150 bp, random breakage will disrupt many amplicon targets and cause qPCR to detect fewer copies than the actual number of plasmid-derived fragments.

For random fragmentation producing 150 bp median fragments from a 7,824 bp plasmid, we can examine the boundary conditions using the smallest (KAN, 63 bp) and largest (SPIKE, 233 bp) amplicons:

- KAN (63 bp): P(intact) = 65.7% → requires 1.52× correction factor
  - Fragmentation destroys 34.3% of KAN amplicons
  - Without correction: 8 ng/dose reported
  - With proper correction: 12.2 ng/dose → FAILS 10 ng regulatory limit
- SPIKE (233 bp): P(intact) ≈ 0% → CANNOT BE QUANTIFIED
  - Amplicon (233 bp) exceeds median fragment size (150 bp)
  - Most fragments are physically too short to contain the complete amplicon
  - Fragmentation to 150 bp destroys the majority of 233 bp SPIKE amplicons

Most critically, even for the smallest amplicon (KAN), applying the required fragmentation correction results in their calculation in exceeding the regulatory limit (12.2 ng >10 ng). For the most biologically relevant sequence (SPIKE), their fragment size distribution makes qPCR quantification essentially impossible, yet they report SPIKE values without acknowledging this fundamental limitation.

### Systematic fragment size bias in primer design

The authors employed vastly different amplicon sizes across their qPCR targets, creating systematic detection bias:

- KAN (kanamycin resistance): 63–93 bp amplicon
- SPIKE (most biologically relevant): 108–233 bp amplicon
- ORI (origin of replication): 78–110 bp amplicon

Given the median fragment sizes of 130–201 bp, the probability that a random fragment contains an intact 233 bp SPIKE amplicon region is dramatically lower than containing a 63 bp KAN region. This creates the false impression that spike-encoding DNA is present at lower levels, when in reality it simply reflects amplicon size bias.

Critical implication: The most biologically concerning sequence (SPIKE) is systematically underestimated relative to antibiotic resistance markers, rendering safety assessments invalid. This fragmentation estimate falsely assumes all regions of the plasmid are equally digested with DNase I. This is noteworthy as McKernan et al., Speicher et al. and Fleming et al. all report 4–6 delta CTs between Spike amplicons and plasmid backbone amplicons where these amplicons are of the similar length (105bp vs. 114bp). McKernan et al. demonstrate this is the result of DNaseI having reduced efficiency on RNA:DNA hybrids and is supported by Sutton et al. and Lenk et al. [5, 6, 25–27].

### Heat-induced DNA fragmentation during sample preparation

For sequencing library preparation, Achs et al. state: “Samples were first heated at 95°C for 10 minutes to release nucleic acids from lipid nanoparticles.” This thermal treatment in aqueous buffer causes extensive DNA fragmentation through:

- Thermal depurination of bases [28–31]
- Hydrolysis at a basic sites
- Phosphodiester bond cleavage

Similarly, for Qubit fluorometry analysis (which uses PicoGreen dye), related work by Speicher et al. used 95°C for 8 minutes prior to fluorometric quantification. Heat treatment causes DNA fragmentation, and Georgiou et al. demonstrated that DNA fragmentation drastically affects PicoGreen-based quantification, with fragmented DNA (<23 kb) underestimated by up to 70% compared to intact DNA [32]. In response to this critique, McKernan et al. reduced the heat treatment to 95°C for 1 minute for their fluorometry protocol to minimize heat-induced fragmentation and reduce the underestimation of artifacts [25, 33].

Critically, Achs et al. used 95°C for 10 minutes during Illumina sequencing library preparation to assess fragment size distributions. However, Illumina library preparation introduces multiple sources of size bias:

- Multiple SPRI (solid phase reversible immobilization) bead cleanup steps select for specific size ranges.
- PCR amplification cycles preferentially amplify shorter fragments
- Cluster generation PCR does not support efficient amplification of fragments >1 kb
- Initial 95°C heat treatment fragments DNA before library construction

In contrast, Speicher, McKernan, and colleagues used Oxford Nanopore Technology (ONT) sequencing for fragment size analysis [5, 25], which:

- Sequences DNA directly with zero amplification.
- Has unlimited read length capability (reads >100 kb routinely achieved).
- Does not require heat treatment for library preparation.
- Preserves native fragment size distributions.

This fundamental difference means that Achs et al.’s fragment size measurements (median 130–201 bp) are artifacts of their Illumina library preparation methodology and do not accurately represent native DNA fragment sizes in the vaccines. Oxford Nanopore sequencing by McKernan et al. and Speicher et al. detected fragments up to 3 kb in their initial analysis [5, 6], and McKernan [25] subsequently detected fragments up to 5.3 kb using improved ONT methods. These findings demonstrate that Achs’s Illumina-based size measurements systematically exclude the largest and potentially most immunogenic DNA fragments.

Notably, Achs et al. clearly possess Oxford Nanopore sequencing capability, as evidenced by their use of Oxford Nanopore RNA controls in their fluorometry experiments. Yet, they chose not to employ this platform for fragment size analysis, instead relying on the amplification-limited and size-biased Illumina platform. This decision is difficult to reconcile with stated goals of accurate contamination assessment.

The methodological inconsistency creates a fatal flaw: fragment sizes measured with heat-treated, amplification-biased Illumina cannot be validly used to interpret DNA quantities or to dismiss contamination concerns. Achs et al. used the wrong platform for accurate fragment size assessment rendering their plasmid equivalent calculations invalid.

### Internal methodological contradiction: Qubit reliability

A striking internal contradiction exists in Achs et al.’s treatment of fluorometry-based quantification. They used Qubit fluorometry (PicoGreen-based) to quantify DNA for titrating samples prior to Illumina sequencing - a necessary step because Illumina sequencing is expensive and requires precise DNA input concentrations. This demonstrates their recognition that fluorometry provides sufficiently accurate quantification for the highly sensitive, single-molecule amplified Illumina platform.

However, when other laboratories (including Konig, Kammerer, Raoult, Wang, McKernan, Speicher, et al.) reported Qubit-based DNA measurements exceeding regulatory limits, Achs et al. dismissed fluorometry as unreliable to quantitate fragmented DNA in mRNA vaccines due to cross-reactivity. This represents a fundamental inconsistency and presents the following statements that cannot both be true:

1. Qubit is reliable to titrate DNA for expensive Illumina sequencing.
2. Qubit is unreliable when it detects DNA levels above regulatory limits of above 10 ng/dose.

One cannot simultaneously rely on a method for one’s own critical measurements while dismissing identical measurements by other laboratories using the same platform. This contradiction suggests methodological choices driven by desired conclusions rather than analytical validity.

### Analytical detection limits exceed regulatory thresholds

Achs et al. employed the Agilent DNF-464 HS Large Fragment Kit for capillary electrophoresis quantification. The manufacturer’s specifications for this platform include both the lower detection limit for DNA smears: 50 pg/μL and an optimal quantification range: 1 ng/μL. However, the regulatory limit for vaccine DNA contamination is 10 ng total DNA per 300 μL dose = 33.3 pg/μL. This leads to a critical gap in methodology. The Fragment Analyzer cannot detect DNA smears (which fragmented plasmid DNA represents) at concentrations below 50 pg/μL, yet the regulatory limit is 33.3 pg/μL. This creates a 1.5-fold “blind spot” where samples could be non-compliant yet reported as “below detection.”

Samples in the range of 33–50 pg/μL would be above the regulatory limit (non-compliant) and below the instrument detection limit (“undetectable”). This renders their claimed “below detection” conclusions meaningless for regulatory assessment. The proper analytical approach would require qPCR or fluorometry with detection limits well below 33 pg/μL.

### Electrokinetic injection matrix effects

A critical flaw in the Fragment Analyzer methodology involves electrokinetic injection incompatibility with the vaccine matrix. Capillary electrophoresis relies on electrokinetic injection (typically 3 kV for 5 seconds) to introduce samples into the capillary. However, Achs et al. analyzed the raw vaccine matrix containing:

1. Salts (buffering agents)
2. Polyethylene glycol (PEG)
3. ALC-0315 (cationic lipid nanoparticle component)
4. 10% Triton X-100 (their stated concentration is ambiguous - unclear if this is final concentration or stock concentration).

The cationic lipid ALC-0315 is particularly problematic for electrokinetic injection. Cationic lipids carry positive charges that directly oppose the electric field used for injection, effectively suppressing DNA migration into the capillary. A 3 kV, 5-second electrokinetic injection (standard conditions for clean DNA samples) would be severely compromised or completely prevented by the presence of cationic lipids in the matrix.

Critically, Achs et al. ran their DNA fragment standards and plasmid controls in clean buffer, not in the vaccine matrix. This means that their standard curves and calibrations were generated under conditions entirely different from their vaccine samples. Their electrokinetic injection conditions were optimized for clean standards and would inject negligible amounts of DNA from the vaccine matrix samples due to cationic lipid suppression.

This creates a systematic underestimation related to the standards for clean buffer and full injection efficiency, and from the vaccine samples in a cationic lipid matrix from a severely suppressed injection. The result of these issues would necessarily lead to vaccine DNA that would appear lower than standards, even at equal concentrations.

The proper approach would require either: (1) preparing standards in matched vaccine matrix, or (2) completely removing all matrix components before analysis. Achs et al. did neither. Their Fragment Analyzer measurements are therefore invalid - not because of detection limits alone, but because the analytical platform fundamentally cannot inject DNA from the vaccine matrix under their stated conditions.

### Short-read sequencing platform bias against long DNA fragments

Achs et al. employed Illumina short-read sequencing, which has well-documented limitations for detecting long DNA fragments including library preparation, which is optimized for 150–250 bp inserts, cluster generation efficiency which declines with smaller with fragment length, and systemic underrepresentation of fragments greater than 800 bp.

Independent analyses using Oxford Nanopore long-read sequencing have detected plasmid DNA fragments exceeding 3 kb in the same vaccine lots [6, 25]. This platform limitation means that Achs et al. would not be able to accurately determine the fragment length and systematically miss the largest and potentially most immunogenic DNA fragments.

Their claim that DNA exists only as “small, degraded fragments” is an artifact of their sequencing platform choice, not a property of the vaccine.

### Failure to account for RNA:DNA hybrid formation

Perhaps most critically, Achs et al. did not consider the formation of RNA:DNA hybrids between residual plasmid DNA and vaccine mRNA. RNA:DNA hybrids are well-documented by-products of in vitro transcription, particularly when transcribing purine-rich sequences, templates containing GAA repeats and using N1-methylpseudouridine base replacements. Importantly, RNA:DNA hybrids pose unique challenges for quality control because standard DNase I treatment, routinely used to remove DNA templates, is highly inefficient at digesting these structures. Sutton et al. demonstrated that the specific activity of DNase I for RNA:DNA hybrids is at least 100-fold lower than for double-stranded DNA [26]. More recently, Lenk et al. from BioNTech explicitly acknowledged this concern stating: “Removal of the dsDNA template from IVT reaction mixes is usually achieved by digestion with DNase I ... The specific activity of DNase I for RNA:DNA hybrids, however, is at least 100-fold below that for dsDNA ... Residual RNA:DNA hybrids may thus be co-isolated together with the single-stranded mRNA” [27].

Recent work by McKernan et al. demonstrates that RNA:DNA hybrids form during vaccine manufacturing [25] and that these hybrids resist standard DNase I digestion. Spike-encoding sequences are enriched 10-100-fold in hybrid form and lipid nanoparticle encapsulation protects hybrids from cellular nucleases. It is well-documented in the published literature that RNA:DNA hybrids activate multiple innate immune pathways beyond cGAS-STING, including TLR3 and RIG-I [34–36].

The biological implications extend beyond immune activation to oncogenic risk. DNA fragments containing SV40 regulatory elements, when protected in hybrid form and delivered via LNPs can enter the nucleus via the SV40 nuclear targeting sequence [13], may sequester p53 through SV40 promoter binding [14] and potentially recruit APOBEC cytidine deaminases causing mutagenic C-to-T transitions through the SV40 enhancer [15]. There is also the risk that SV40 fragments could integrate into the genome [16]. Given there are human UTRs in the vaccines both homologous and non-homologous end joining need to be considered.

Achs et al.’s entire framework assumes that that DNA present in the mRNA vaccines are naked and degraded DNA fragments with limited biological activity. The RNA:DNA hybrid mechanism, combined with the oncogenic potential of SV40 sequences explicitly acknowledged in Moderna’s patents [20–22] completely invalidates this assumption. Yet, they made no attempt to measure or account for hybrid formation, nor did they address the specific oncogenic risks posed by SV40-containing DNA fragments. In fact, their plasmid DNA depictions in their supplement also omit any annotation of the SV40 components documented in the BioNTech vaccine.

### Lack of size-dependent recovery validation

While Achs et al. reported using a 25–700 bp ladder as spike-in control, they provided only overall recovery percentages (60–80% for phenol-chloroform, 90–95% for magnetic beads). They did not report recovery efficiency by fragment size, whether 25 bp fragments were recovered at the same efficiency as 700 bp fragments, or the raw data showing ladder recovery across the size range. Many DNA extraction kits preferentially retain longer fragments while losing those <200 bp. Without demonstrating size-independent recovery, the overall percentages could mask preferential loss of smaller fragments, further biasing results.

### Ignored preprint critiques and undisclosed conflicts of interest

Many of the methodological flaws documented in this critique were previously raised during the preprint stage of Achs et al.’s work. Comments were posted on the Research Square preprint server (https://www.researchsquare.com/article/rs-7340318/v1) in September 2nd, 2025, and detailed critiques published at https://zenodo.org/records/17409084 [33] identified multiple critical issues including fragmentation correction requirements, DNA purification controls, flawed primer design, flawed qPCR inhibition testing detection, and the requirement for proper conflict of interest disclosure.

Despite these valid critiques being publicly available prior to final publication, Achs et al. chose to ignore most of these recommendations. Most concerning was their failure to disclose that the authors are employed at a vaccine research institution with documented funding from mRNA vaccine manufacturers. This represents a clear conflict of interest that should have been disclosed per standard academic publishing practice.

One critique they did nominally address was the recommendation to validate DNA recovery across fragment sizes using a 10 bp ladder rather than their initial 1 kb standard. In response, they claimed to have used a 25–700 bp ladder (GeneRuler Low Range DNA Ladder from the same manufacturer (Thermo Fisher Scientific), advertised on the same product page as the 10 bp ladder). However, suspiciously, none of their reported recovery values changed between preprint and publication (60–80% for phenol-chloroform, 90–95% for magnetic beads), suggesting this was a post-hoc modification of the methods section to address reviewer concerns rather than actual experimental validation.

This pattern of ignoring substantive technical critiques while making superficial methodological claims without corresponding data changes raises serious questions about the scientific integrity of the publication process.

### Process change and DAM/DCM methylation effects

An important contextual issue not addressed by Achs et al. is the change from Process 1 to Process 2 in Pfizer’s BNT162b2 vaccine manufacturing, which introduced E. coli-produced plasmid DNA templates containing bacterial DAM (N6-methyladenine) and DCM (5-methylcytosine) methylation patterns. This manufacturing change has been documented in regulatory submissions and scientific correspondence [37].

Critically, bacterial DNA methylation patterns, particularly CpG motifs with methylation, are known to be more immunostimulatory through the cGAS-STING pathway compared to unmethylated DNA [38]. This means that even if Achs et al. had accurately quantified residual DNA contamination, they failed to account for the enhanced immunogenicity of bacterially methylated plasmid DNA present in commercial vaccine lots.

The Process 1 to Process 2 change as part of upscaling the manufacturing process, represents an additional variable affecting vaccine immunogenicity that Achs et al. completely overlooked. Their conclusions about DNA contamination safety cannot be extrapolated to commercial vaccine lots without considering the impact of bacterial methylation patterns on innate immune activation.

## DISCUSSION

The motivation for this technical review stems from Achs et al. being cited by regulatory agencies and manufacturers as evidence that DNA contamination levels in mRNA vaccines are within acceptable limits. Any publication with significant policy implications impacting the global administration of a novel, liability-free, and often mandated product, should welcome public scrutiny of methodological flaws that may bias results. Documenting these flaws serves the scientific community and public health and does not spread “vaccine hesitancy”. However, the failure to address the concerns may in fact have that unintended consequence. We conclude that the cumulative effect of the methodological flaws in Achs et al. creates systematic underestimation of DNA contamination through multiple independent mechanisms including:

1. 1.5-fold underestimation from missing fragmentation correction (for KAN).
2. >10-fold underestimation of SPIKE sequences from amplicon size bias.
3. Invalid fragmentomics due to heat-induced degradation.
4. Unknown underestimation from Fragment Analyzer detection limits.
5. Complete exclusion of >1 kb fragments from sequencing platform limitations.
6. Failure to measure hybrid-protected spike DNA.
7. Dismissal of undisclosed fragmented DNAs potential functionality regarding cGAS-STING activation, mammalian nuclear targeting sequences, p53 interactions, somatic hypermutability elements, mammalian promoters (ColE1 proximal to spike), enhancers and origins of replication.

These are not minor technical issues, but fundamental design flaws that make the study incapable of detecting the contamination that it claims to assess. The convergence of multiple biases in the same direction (underestimation) raises concerns about whether methodological choices were made to achieve predetermined conclusions.

### Comparison to proper methodology

A rigorous assessment of vaccine DNA contamination would require qPCR using fragment-appropriate molecular weights for mass conversion, amplicon sizes optimized for detected fragment length distributions, sample preparation avoiding thermal degradation, analytical platforms with detection limits below regulatory thresholds, long-read sequencing to capture full size distribution, specific measurement of RNA:DNA hybrid content using RNase A or DNase I-XT and size-dependent recovery validation for all extraction methods. Studies employing these approaches have consistently detected DNA contamination exceeding regulatory limits [5–7, 12], in stark contrast to Achs et al.’s conclusions.

## CONCLUSIONS

Achs et al. claimed to provide a “systematic analysis” demonstrating “no excessive DNA impurities” in mRNA vaccines. However, their methodology contains multiple systematic flaws that invariably bias the results toward an underestimation of DNA contamination. The failure to apply fragmentation correction factors, combined with amplicon size bias, thermal degradation, detection limit gaps, electrokinetic injection suppression, platform limitations, and failure to measure RNA:DNA hybrids, compounds to create systematic underestimation.

These are not subtle technical disagreements - but fundamental methodological failures - that render the study’s conclusions invalid for regulatory assessment. The scientific community and regulatory agencies should not rely on this publication for safety evaluations of residual DNA detected in mRNA vaccines.

Proper assessment of vaccine DNA impurities requires methodologies specifically designed to detect and quantify the biologically relevant forms of residual DNA, including RNA:DNA hybrids and long fragments, using analytical platforms with appropriate sensitivity. Until such studies are performed with rigorous methodology, the question of DNA contamination remains unresolved.

## Abbreviations

bp: base pairs
cGAS-STING: cyclic GMP-AMP synthase-stimulator of interferon genes
COVID-19: Coronavirus Disease 2019
DNA: deoxyribonucleic acid
dsDNA: double-stranded DNA
EMA: European Medicines Agency
FDA: Food and Drug Administration
KAN: kanamycin (resistance gene)
kb: kilobase pairs
LNP: lipid nanoparticle
mRNA: messenger RNA
MW: molecular weight
ng: nanogram(s)
ONT: Oxford Nanopore Technology
ORI: origin of replication
PCR: polymerase chain reaction
PEG: polyethylene glycol
pg: picogram(s)
qPCR: quantitative polymerase chain reaction
RNA: ribonucleic acid
SPRI: solid phase reversible immobilization
SV40: Simian Virus 40
WHO: World Health Organization
μL: microliter(s)
